# Effects of hydroxyapatite coating on survival of an uncemented femoral stem: A Swedish Hip Arthroplasty Register study on 4,772 hips

**DOI:** 10.3109/17453674.2011.597699

**Published:** 2011-09-02

**Authors:** Stergios Lazarinis, Johan Kärrholm, Nils P Hailer

**Affiliations:** ^1^Department of Orthopaedics, Institute of Surgical Sciences, Uppsala University Hospital, Uppsala; ^2^Department of Orthopaedics, Institute of Surgical Science, Sahlgrenska University Hospital, Göteborg University, Mölndal, Sweden; Correspondence: lazarinis.stergios@surgsci.uu.se

## Abstract

**Background and purpose:**

Hydroxyapatite (HA) is widely used as a coating for uncemented total hip arthroplasty components. This has been suggested to improve implant ingrowth and long-term stability. However, the evidence behind the use of HA coating on femoral stems is ambiguous. We investigated survival of an uncemented, tapered titanium femoral stem that was available either with or without HA coating (Bi-Metric).

**Patients and methods:**

The stem had been used in 4,772 total hip arthroplasties (THAs) in 4,169 patients registered in the Swedish Hip Arthroplasty Register between 1992 and 2009. 59% of the stems investigated were coated with HA and 41% were uncoated. Kaplan-Meier survival analysis and a Cox regression model with adjustment for age, sex, primary diagnosis, and the type of cup fixation were used to calculate survival rates and adjusted risk ratios (RRs) of the risk of revision for various reasons.

**Results:**

The 10-year survival rates of the HA-coated version and the uncoated version were about equal when we used revision for any reason as the endpoint: 98% (95% CI: 98–99) and 98% (CI: 97–99), respectively. A Cox regression model adjusting for the covariates mentioned above showed that the presence of HA coating did not have any influence on the risk of stem revision for any reason (RR = 1.0, 95% CI: 0.6–1.6) or due to aseptic loosening (RR = 0.5, CI: 0.2–1.5). There was no effect of HA coating on the risk of stem revision due to infection, dislocation, or fracture.

**Interpretation:**

The uncemented Bi-Metric stem showed excellent 10-year survival. Our findings do not support the use of HA coating on this stem to enhance implant survival.

It is generally believed that coating of total hip arthroplasty (THA) components with hydroxyapatite (HA) improves implant ingrowth and long-term stability. Thus, a large number of prostheses designed for uncemented hip arthroplasty are coated with HA. In Europe, some manufacturers mainly or exclusively market uncemented hip prostheses with such a coating.

The evidence behind the use of HA is ambiguous, however. Several reports on smaller series have described varying outcomes after the use of HA-coated cups or stems. Good or even excellent results were found after the use of some HA-coated implants, with survival rates close to 100% when using revision or impending revision for aseptic loosening as the endpoint (Oosterbos et al. 2001, Capello et al. 2003, Shah et al. 2009). On the other hand, mediocre to obviously inferior results of HA-coated hip arthroplasty components have also been reported (Havelin et al. 2000, Reikerås and Gunderson 2002, Cheung et al. 2005, Kim et al. 2006). A large Danish registry analysis on uncemented hip implants found that HA coating did not reduce the risk of revision in patients younger than 70 years of age (Paulsen et al. 2007). In a recent analysis based on data from the Swedish Hip Arthroplasty Register, we found that HA coating of acetabular cups could even increase the risk of revision due to aseptic loosening (Lazarinis et al. 2010).

In this study, we analyzed survival of uncemented femoral stems in the Swedish Hip Arthroplasty Register that were used either with or without HA coating. Our main hypothesis was that HA coating influences the risk of stem revision for any reason, which was our primary endpoint. Secondary endpoints were stem revision due to aseptic loosening, infection, fracture, or dislocation.

## Patients and methods

### Sources of data and study population

The Swedish Hip Arthroplasty Register (2009) was the source of our data. All orthopedic units in Sweden that perform total hip arthroplasty, both public and private, are included in the Register. All reoperations (secondary operation of the hip) and revisions (exchange or removal of any of the components) have been continuously reported by all operating units in Sweden since 1979. From 1992 onwards, implants inserted during THA have been linked to the personal ID number, and information gathered includes the type of implant, fixation, and technical details such as HA coating. The Swedish ID number enables registration of changes of address and dates of emigration or death, information that is necessary in order to perform survival analyses. The Swedish Hip Arthroplasty Register has been repeatedly validated (Söderman et al. 2000, 2001).

The only uncemented femoral stem that was available with or without HA coating (according to the Swedish Hip Arthroplasty Register) during the time period 1992–2009 was the Bi-Metric prosthesis (Biomet Inc., Warsaw, USA). This stem is an uncemented, tapered implant made of titanium alloy (Ti-6Al-4V) where the proximal third has a plasma-sprayed, titanium alloy porous coating with a mean pore size of 300 μm. The distal part has a textured surface with a roughness of 6.9 μm. In the HA-coated version, the proximal, porous-coated part of the stem is covered with a plasma-sprayed HA layer. The HA coating has a thickness of 40–70 mm, a crystallinity of 50–70%, and a purity of greater than 95%, although the manufacturer has stated that changes in the composition of the HA coating have been made over time. We identified 4,772 THAs in 4,169 patients in whom the Bi-Metric femoral stem had been used.

### Statistics

Follow-up started on the day of primary THA and ended on the day of revision, death, emigration, or December 31, 2009, whichever came first. Kaplan-Meier survival analysis was performed on the entire study cohort with HA coating as the independent factor, and stem revision for any reason or due to aseptic loosening as the endpoints. The log-rank test (Mantel-Cox) was used to investigate differences between groups, and p-values < 0.05 were considered significant.

A Cox proportional hazards model was applied in order to examine the influence of HA coating on the relative risk (RR) of stem revision, with 95% confidence intervals (CIs), adjusting for the covariates age (≤ 49, 50–59, 60–75, and > 75 years), sex, primary diagnosis (primary osteoarthritis (OA) or other diagnoses), and type of cup fixation (cemented or uncemented). These covariates were entered into the regression model and risk ratios were calculated for each variable, mutually adjusted for all other covariates. Adjusted risk ratios were calculated for stem revision for any reason or due to aseptic loosening, infection, dislocation, or fracture. The assumption of proportional hazards was investigated by hazard function plots and log-minus-log plots of all covariates. There was no sign of insufficient proportionality in the hazard functions, and log-minus-log plots ran parallel for all covariates.

The inclusion of both joints in bilaterally operated patients has been proposed to lead to dependency issues. Thus, we performed a separate analysis of all joints (4,772 hips in 4,169 individuals in the Cox regression model), and 4,169 joints after excluding the second hip in bilaterally operated patients. The results were not statistically significantly different when all hips or only 4,169 hips were included (data not shown). All analyses were performed using PASW software (version 18.0).

## Results

### Characteristics of the study population

The numbers of males and females were similar. The largest number of THAs was found in the age group between 50 and 59 years, and primary osteoarthritis was the most common preoperative diagnosis (Table 1). Different types of cemented and uncemented cups were combined with the stems (Tables 2 and 3). By 2009, 72 (1.5%) of all 4,772 stems had been revised, 14 (0.3%) due to aseptic loosening, 28 (0.6%) due to fracture, 12 (0.3%) due to deep infection, and 8 (0.2%) due to dislocation. The mean follow-up time for all stems was 4.5 (SD 4.4) years—4.8 (SD 4.0) years for the HA-coated stems and 4.0 (SD 4.8) years for the uncoated stems.

**Table 1.** Characteristics of the 4,169 patients studied in the Swedish Hip Arthroplasty Register (1992–2009)

	+ HA		– HA		Total	
	n	%	n	%	n	%
Sex						
Male	1,290	53	735	42	2,025	49
Female	1,131	47	1,013	58	2,144	51
Age (years)						
0–49	497	20	378	22	875	21
50–59	1,005	42	691	39	1,696	41
60–75	795	33	600	34	1,395	33
> 75	124	5	79	5	203	5
Primary diagnosis						
Primary OA	2,140	88	1,470	85	3,610	87
Other	279	12	265	15	544	13

In 17 hips (15 patients), the primary diagnosis was unknown or not reported to the register. In bilaterally operated patients, age and diagnosis at the time of the first operation were used to calculate frequencies. The category “Other” under “Primary diagnosis” includes the diagnoses of rheumatoid arthritis and related disorders, cervical neck fracture, pediatric hip disease, and further diagnoses.

**Table 2.** Distribution of cemented and uncemented cups

	Stems					
	+ HA		– HA		Total	
	n	%	n	%	n	%
Cemented	1,482	53	809	41	2,291	48
Uncemented	1,318	47	1,163	59	2,481	52
Total	2,800	100	1,972	100	4,772	100

**Table 3.** Distribution of the 7 most frequently used cups combined with the stem investigated

	Bi-Metric stem			
	+ HA		– HA	
	n	%	n	%
Biomet Müller	410	15	112	6
Trilogy HA	330	12	252	13
Romanus HA	276	10	87	4
ZCA XLPE	272	10	170	9
Lubinus	262	9	79	4
Charnley	239	8	199	10
Romanus	136	5	243	12
Others	875	31	830	42
	2,800	100	1,972	100

### Risk of stem revision for any reason

**Figure 1.**
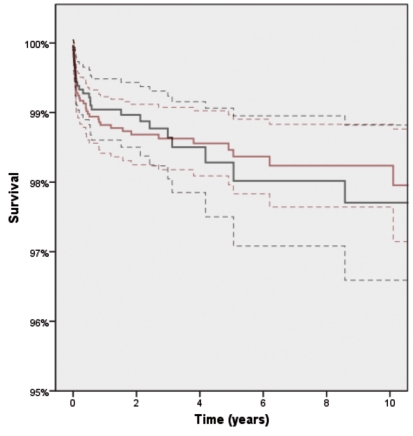
Kaplan-Meier analysis with hydroxyapatite (HA) coating as the independent factor and stem revision for any reason as the endpoint. 10-year survival was 98.2% (CI: 97.6–98.8) for the HA-coated stems (red) and 97.7% (CI: 96.5–98.9) for the uncoated stems (black). The dashed lines represent 95% CIs for the 2 groups of stems (p = 1.0).

Kaplan-Meier analysis showed a similar 10-year survival of 98% (CI: 98–99) for the HA-coated stems and 98% (CI: 97–99) for the uncoated stems, with stem revision for any reason as the endpoint (Figure 1).

In a Cox regression model the crude RR of HA coating for the risk of stem revision for any reason was 0.99 (CI: 0.62–1.6) without adjustment for covariates. Likewise, HA coating did not affect the risk of stem revision for any reason after adjustment for all other covariates, with an adjusted RR of 1.0 (CI: 0.62–1.6) (Table 4). The only variable that had an influence on the risk of stem revision was the type of primary diagnosis: patients with diagnoses other than osteoarthritis ran a higher risk of stem revision (Table 4). This effect was mainly attributable to the facts that (1) patients operated for cervical neck fracture had a 5-fold increased risk of stem revision (CI: 2.1–13; p < 0.001), and (2) patients operated due secondary arthritis after pediatric hip disease had a 3-fold increased risk of stem revision (CI: 1.4–7; p = 0.006) when compared to patients with osteoarthritis. However, the numbers of revisions in these subgroups were small.

**Table 4.** Relative risk (RR) of stem revision for any reason

Endpoint:	No. of hips	No. of revisions	Adjusted RR **^a^** (95% CI)	p-value
Coating				
– HA	1,972	29	1.0 (ref) **^b^**	
+ HA	2,800	43	1.0 (0.62–1.62)	1
Sex				
Male	2,316	35	1.0 (ref)	
Female	2,456	37	0.89 (0.55–1.42)	0.6
Primary diagnosis				
Primary OA	4,146	52	1.0 (ref)	
Other **^c^**	609	20	2.77 (1.57–4.87)	< 0.001
Age				
0–49	1,033	18	1.0 (ref)	
50–59	1,977	27	1.16 (0.61–2.19)	0.7
60–75	1,551	23	1.56 (0.77–3.17)	0.2
> 75	211	4	2.41 (0.74–7.81)	0.1
Cup fixation				
Cemented	2,291	32	1.0 (ref)	
Uncemented	2,481	40	0.94 (0.55–1.61)	0.8

**^a^**A Cox proportional hazards model was used where covariates (HA coating, sex, primary diagnosis, age, and type of cup fixation) were entered in the regression model and risk ratios were mutually adjusted for all covariates. Adjusted risk ratios (RRs) were calculated for revision for any reason.

**^b^**ref: reference group.

**^c^**The category “Other” under ”Primary diagnosis” includes the diagnosis of rheumatoid arthritis and related disorders, cervical neck fracture, pediatric hip disease, and further diagnoses.

### Risk of stem revision due to aseptic loosening or for other reasons

**Figure 2.**
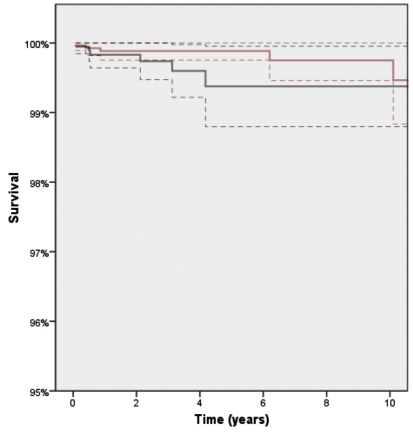
Kaplan-Meier analysis with hydroxyapatite (HA) coating as the independent factor and stem revision due to aseptic loosening as the endpoint. 10-year survival was 99.8% (CI: 99.6–100) for the HA-coated stems (red) and 99.4% (CI: 98.8–100) for the uncoated stems (black). The dashed lines represent 95% CIs for the 2 groups of stems (p = 0.23).

Kaplan-Meier analysis with HA coating as the independent factor and stem revision due to aseptic loosening as the endpoint showed that there was no difference in survival between stems coated with HA and uncoated stems. The 10-year survival was 99.8% (CI: 99.6–100) for the HA-coated stems and 99.4% (CI: 98.8–100) for the uncoated stems (p = 0.2) (Figure 2). When we adjusted for the covariates described above in the Cox regression model, the presence of HA coating did not affect the risk of stem revision due to aseptic loosening (RR = 0.5, CI: 0.17–1.5) (Table 5).

**Table 5.** Relative risk (RR) of stem revision due to aseptic loosening, infection, dislocation, or fracture

Endpoint:	No. of hips	No. of revisions	Adjusted RR **^a^** (95% CI)	p-value
Aseptic loosening				
– HA	1,972	8	1.0 (ref) **^b^**	
+ HA	2,800	6	0.5 (0.17–1.5)	0.2
Infection				
– HA	1,972	5	1.0 (ref)	
+ HA	2,800	7	0.8 (0.25–2.6)	0.7
Dislocation				
– HA	1,972	2	1.0 (ref)	
+ HA	2,800	6	2.6 (0.52–13.2)	0.3
Fracture				
– HA	1,972	10	1.0 (ref)	
+ HA	2,800	18	1.2 (0.56–2.7)	0.6

**^a^**A Cox proportional hazards model was used to investigate the influence of HA coating on the relative risk (RR) of stem revision (with 95% CIs), adjusted for the covariates age (0–49, 50–59, 60–75, and > 75), sex, primary diagnosis (primary osteoarthritis (OA) and other diagnoses), and type of cup fixation (cemented or uncemented). Adjusted risk ratios (RRs) were calculated for revision due to aseptic loosening, infection, dislocation, or fracture.

**^b^**ref: reference group.

The presence of HA coating was not found to have any influence on the the risk of stem revision due to infection, dislocation, or fracture in a Cox regression model with adjustment for age, sex, primary diagnosis, and the type of cup fixation (Table 5).

## Discussion

### Influence of HA coating on stem survival

The Bi-Metric stem is more widely used with HA coating than without, and this probably reflects that HA coating is believed to improve stem fixation and long-term stability. Indeed, a retrieval study on this stem found that there was more bone ingrowth around stems with HA coating than around those without (Coathup et al. 2001). However, a review of the literature on HA-coated uncemented femoral components failed to show an improvement in long-term stability (Chambers et al. 2007). Similarly, a large Danish registry analysis on survival of HA-coated hip implants indicated that the femoral stem under investigation did not benefit from the use of HA coating (Paulsen et al. 2007).

Randomized studies on smaller cohorts have reported medium-term results on other femoral stems available with or without HA coating in bilaterally operated patients. In several studies, HA-coated titanium stems were implanted on one side and identical stems without an HA coating were inserted on the opposite side. It was found that HA coating did not influence radiological results or clinical performance of these stems in the medium-term (Kim et al. 2003, Park et al. 2003). Recently published meta-analyses of HA-coated femoral stems in primary THA have also supported the notion that HA coating does not improve the survival of uncemented stems (Gandhi et al. 2009, Goosen et al. 2009).

### Performance of the Bi-Metric stem

The tapered titanium femoral stem investigated in our study had excellent 10-year survival rates. Other studies on smaller numbers of patients who had received this stem have shown survival rates of between 95% and 100% at 10 years (Isaac et al. 2007, Davies et al. 2010). A low revision rate of the Bi-Metric stem was also found in a large Finnish registry analysis with 10-year survival of 96% based on aseptic loosening as the endpoint (Eskelinen et al. 2006). Similar results have been found in patients under the age of 55 years, i.e. a group with higher risk of early revision (Puolakka et al. 1999), and in patients over 55 years (Mäkelä et al. 2010).

Stress shielding due to distal load transfer between the stem and the femur can lead to excessive loss of proximal bone mineral density, especially in Gruen zones 1 and 7. This might result in implant subsidence, periprosthetic fractures, or loosening (Otani and Whiteside 1992). Proximal bone loss around uncemented stems is a well-known phenomenon (Panisello et al. 2009, Pitto et al. 2010). A literature review of retrospective and prospective studies found that decreasing proximal bone density will persist for at least 1 year after implantation (Kröger et al. 1998). Several authors have pointed out that more or less pronounced periprosthetic proximal bone loss also occurs after the use of the Bi-Metric stem, after both primary and revision operations (Bodén et al. 2006, Sköldenberg et al. 2006, Adolphson et al. 2009). However, this phenomenon does not appear to influence the long-term performance of the stem.

We found that the risk of stem revision was higher in patients who received this implant due to cervical neck fracture or due to secondary arthritis after pediatric hip disease.These findings are in agreement with reports from the Swedish Hip Arthroplasty Register indicating that patients operated with a total hip prosthesis due to cervical neck fracture or secondary arthritis are at higher risk for prostheses loosening than those operated due to primary osteoarthritis (Swedish Hip Arthroplasty Register 2009).

### Confounding factors

Inferior performance of uncemented cups compared to cemented cups has been reported (Havelin et al. 2000, Hailer et al. 2010). Liner wear, osteolysis, loosening, and early revision of uncemented cups could affect the survival of the stems that were combined with uncemented cups. Because of this potential bias, the type of cup fixation (cemented or uncemented) was introduced as a covariate. However, the regression model indicated that the type of cup fixation did not influence the risk of revision of the stem for any reason as the endpoint (Table 4).

It could be argued that the association of one group of stems, either HA-coated or uncoated, with specific cups of inferior performance could distort stem survival in that group. For instance, the frequent use of cups with higher than average risk of osteolysis could lead to inferior long-term results on the stem side also. An increased amount of polyethylene debris from the cups could finally result in femoral osteolysis and stem revision (Puolakka et al. 1999, Swedish Hip Arthroplasty Register 2009). However, our analysis of the various cups combined with the 2 types of Bi-Metric stems indicates that this was not the case. The distribution of cups combined with two types of stems varied substantially, but there seemed to be no obvious predominance of cups with inferior performance in any of the groups (Table 3). For example, 15% of the HA-coated stems and 16% of the uncoated stems were combined with the Romanus cup, a cup associated with high revision rates (Swedish Hip Arthroplasty Register 2009) mainly because of osteolysis and wear.

Several other covariates with a possible influence on stem survival were also investigated. The type of hospital of primary arthroplasty had no statistically significant influence on the risk of stem revision (data not shown). Some other possible confounding factors such as medications that are known to influence bone metabolism (e.g. steroids, non-steroidal anti-inflammatory drugs, bisphosphonates) were not recorded in the Swedish Hip Arthroplasty Register. The same applies to medical conditions that have an indirect influence on implant survival, such as overweight, diabetes mellitus, or disorders of lipid metabolism.

### Statistical considerations

Our findings indicate that there was excellent 10-year survival of the stem under investigation, with very few stem revisions recorded in the entire cohort (72 stems revised out of 4,772). Implicitly, the number of revisions is even smaller when subgroups of patients are analyzed, a problem that is illustrated by the analysis of the variable “preoperative diagnosis” (see above). Interpretation of our statistical analyses becomes even more difficult when the risk of stem revision due to aseptic loosening is considered, because only 14 stem revisions due to aseptic loosening occurred in the entire study cohort. Thus, absence of statistically significant effects of HA coating on stem survival does not necessarily imply that HA coating has no effect; i.e., the analysis is open to a type-II error. On the other hand, any potential differences between groups will be so small that their clinical relevance can be questioned.

### Conclusion

Our results derived from registry data on 4,772 hips show that there is no difference in stem survival between uncemented Bi-Metric stems with and without HA coating. Our findings agree with the currently available literature on the subject, and do not support the idea that HA coating improves the long-term survival of well-functioning uncemented femoral stems.
